# Possible Combined Effects of Plasma Folate Levels, Global DNA Methylation, and Blood Cadmium Concentrations on Renal Cell Carcinoma

**DOI:** 10.3390/nu15040937

**Published:** 2023-02-13

**Authors:** Chao-Yuan Huang, Wei-Jen Chen, Hui-Ling Lee, Ying-Chin Lin, Ya-Li Huang, Horng-Sheng Shiue, Yeong-Shiau Pu, Yu-Mei Hsueh

**Affiliations:** 1 Department of Urology, National Taiwan University Hospital, College of Medicine National Taiwan University, Taipei City 110, Taiwan; 2 Department of Medicine, Section of Epidemiology and Population Sciences, Baylor College of Medicine, Houston, TX 77030, USA; 3 Department of Chemistry, Fu Jen Catholic University, New Taipei City 242, Taiwan; 4 Department of Family Medicine, Wan Fang Hospital, Taipei Medical University, Taipei City 110, Taiwan; 5 Department of Family Medicine, School of Medicine, College of Medicine, Taipei Medical University, Taipei City 110, Taiwan; 6 Department of Occupational Medicine, Wan Fang Hospital, Taipei Medical University, Taipei City 110, Taiwan; 7 Department of Public Health, School of Medicine, College of Medicine, Taipei Medical University, Taipei City 110, Taiwan; 8 Department of Chinese Medicine, College of Medicine, Chang Gung University, Taoyuan City 333, Taiwan

**Keywords:** 5-Methyl-2-deoxycytidine, folate, vitamin B_12_, cadmium, renal cell carcinoma

## Abstract

Epigenetic effects of environmental pollutants may be related to carcinogenesis. This study aimed to explore the association between the global DNA methylation marker: 5-methyl-2-deoxycytidine (5mdC) and renal cell carcinoma (RCC), and further investigated whether plasma folate and vitamin B_12_ levels and 5mdC modified the association between blood cadmium concentrations and RCC. We recruited 174 RCC patients and 673 non-RCC controls. Blood cadmium concentrations, plasma folate and vitamin B_12_ levels were measured. The amount of 5mdC in the DNA sample was expressed as percentages of the total cytosine content. An increase of 5mdC (%) and plasma folate and vitamin B_12_ levels were associated with decreasing odds ratio (OR) of RCC. Although plasma folate levels were not directly associated with 5mdC (%), a combined effect was observed with the odds of low plasma folate levels and low 5mdC (%) were greater among RCC patients compared to controls (OR (95% confidence interval, CI) = 11.86 (5.27–26.65)). Additionally, we observed that the odds of low plasma folate and high blood cadmium levels were greater among RCC patients than in controls (OR (95% CI): 8.15 (1.39–7.13)). This study provides suggestive evidence that plasma folate levels may modify the associations between 5mdC (%) or blood cadmium concentrations and RCC.

## 1. Introduction

Renal cell carcinoma (RCC) is the most common type of kidney cancer, accounting for 85% of kidney tumors, with a male-to-female ratio of 1.7:1 [1]. As the sixth most common cancer in men and tenth in women worldwide, RCC causes a significant disease burden [1]. The incidence of RCC is still increasing in most countries, while mortality trends have stabilized in many highly developed countries [2]. The age-standardized incidence rates of RCC in Taiwan in 2002 and 2012 were 3.39/10^6^ and 5.09/10^6^, respectively [3]. RCC risk factors such as smoking, obesity, and comorbidities (e.g., diabetes, hypertension, and urolithiasis) are well recognized [4], and exposure to environmental contaminants such as cadmium may also increase the risk of RCC [5,6]. Exposure to cadmium is mainly from rice, which is the Taiwanese staple food [7]. Additionally, other sources such as air pollution from industrial and traffic emissions [8] and smoking [9] can contribute to cadmium exposure. However, the associations of folate and vitamin B_12_, essential nutrients that involve in one-carbon metabolism [10], with RCC have remained inconsistent in previous studies [11,12,13].

A recent study suggested epigenetic parameters as biomarkers reflecting recent and past pollution burdens [14]. DNA methylation is an epigenetic marker sensitive to environmental factors [15]. It is one of the most studied epigenetic modifications, where a methyl group is added to the fifth carbon of the DNA base, cytosine, at CpG dinucleotides. DNA methylation patterns are associated with gene expression and cellular integrity, and genome-wide hypomethylation may be associated with chromosomal instability and oncogenic expression [16]. The global loss of DNA methylation is a feature of cancer tissues [17]. Measuring the ratio of 5-methyl-2′deoxycytidine (5mdC) to 2′-deoxyguanine (dG) (5mdC (%)), assuming (dG) = (5mdC) + (2′-deoxycytidine, dC), provides an estimate of global DNA methylation levels [18,19]. At present, there are few studies using 5mdC (%) as a global DNA methylation marker to explore its relationship with cancer, especially RCC. Only one American study found that global DNA hypomethylation measured by 5mdC (%) was associated with RCC [20]. Furthermore, this study found that the association between hypomethylation and increased risk of RCC appeared to be in males, younger age individuals, non-smokers, and individuals with a family history of other cancers at advanced stages.

Evidence has suggested that global DNA methylation is associated with exposure to cadmium, and global DNA methylation may also be changed according to folate and vitamin B_12_ levels. In vivo research observed that global DNA methylation levels significantly decreased with increasing cadmium concentration in rats [21]. A meta-analysis of randomized controlled trials showed that folate supplementation alone or in combination with vitamin B_12_ significantly increased global DNA methylation [10]. Exploring the role of global DNA methylation and plasma folate and vitamin B_12_ on RCC may provide a better understanding of molecular mechanisms underlying the association between exposure to cadmium and RCC. Therefore, the aim of this study was to investigate the associations of 5mdC (%) and plasma folate and vitamin B_12_ levels with RCC. Furthermore, we explored whether 5mdC (%) and plasma folate and vitamin B_12_ levels could modify the association between blood cadmium concentration and RCC.

## 2. Materials and Methods

### 2.1. Study Subjects

The present study was a clinical-based case–control study. A total of 174 patients with pathologically confirmed RCC and 673 age- and sex-matched individuals without RCC or any other malignancy were recruited as controls from our previous study [5]. In RCC patients, approximately 70% had grade II or III tumors, including 112 clear cell, 17 papillary, 17 chromophobe, 1 sarcoma, and 4 “other” cases; cell type information was not available for 23 of these. All study participants provided written informed consent before the questionnaire interviews and specimen collection. This study was approved by the Institutional Review Board of National Taiwan University Hospital.

### 2.2. Questionnaire Interview and Bio-Specimen Collection

Standardized questionnaire interviews, questionnaire information, and blood sample collection methods have been previously described [5]. Peripheral blood samples of 5–8 mL were collected using an EDTA vacuum syringe. The plasma was separated for the determination of folate and vitamin B_12_ levels, and the buffy coat was separated for DNA extraction and 5mdC measurement.

### 2.3. Measurements of Plasma Folate and Vitamin B_12_ Levels and Blood Cadmium Concentrations

The methods used for measuring plasma vitamin B_12_ and folate levels are described in detail in our previous study [22]. Concentrations of blood cadmium were quantified by inductively coupled plasma mass spectrometry, as described previously [23]. The determination method, detection limits, and reliability of all measurements, and recovery rate and standard reference materials of each measurement are summarized in Supplementary Table S1.

### 2.4. Global DNA Methylation Marker, 5mdC, Measurement

**Figure 1:**
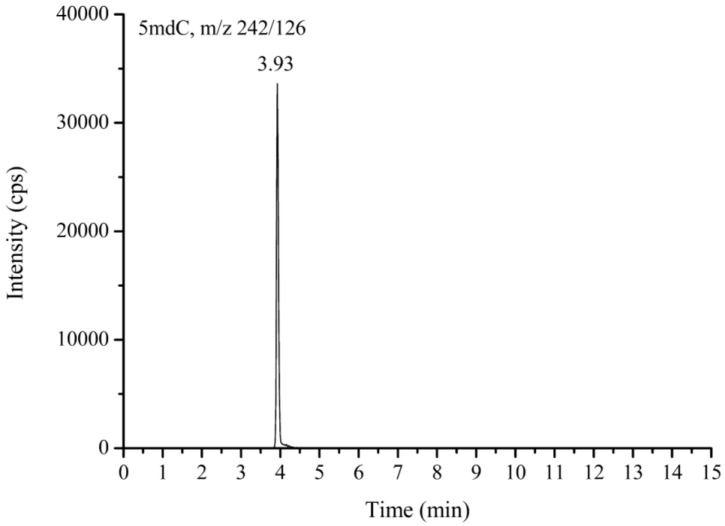
LC-MS/MS chromatogram of reference compounds using multiple reaction monitor mode. 10 ng/mL 5mdC were dissolved in 5% Methanol + 0.1% formic acid.

Proteinase K, phenol, and chloroform digestions were used for DNA extraction. The levels of 5mdC were quantified using high-performance liquid chromatography (Agilent 1260VL, Agilent Technology, Santa Clara, CA, USA) equipped with an API 3000™ triple quadrupole mass spectrometer (AB SCIEX, Concord, ON, Canada). The detailed protocol of the analysis has been described previously [18,19]. The 5mdC level present in the DNA sample was expressed as a percentage of the total cytosine content (methylated and non-methylated). The DNA methylation level was expressed as either (5mdC)/(5mdC + dC) without internal standard adjustments or (5mdC)/(dG) with adjustment to the respective ^15^N-labeled internal standards, where dG is an internal standard that allows a simpler and accurate determination of dC methylation and avoids the use of expensive isotope-labeled internal standards [18,19]. Figure 1 displays the chromatogram of liquid chromatography-tandem mass spectrometry (LC-MS/MS) for detecting reference compounds of 5mdC.

### 2.5. Statistical Analysis

Continuous and categorical variables were summarized using the median (first and third quartile) and frequency (percentage), respectively. To compare the variables between RCC patients and controls, the Wilcoxon rank-sum test and chi-square test were performed for the continuous and categorical variables, respectively. Multivariable linear regression models were used to examine the associations of plasma folate and vitamin B_12_, blood cadmium concentrations, and 5mdC (%) each other, adjusted for covariates. Multivariable logistic regression models were conducted to estimate the OR and 95% confidence intervals (CI), and to assess the associations of 5mdC (%), plasma folate and vitamin B_12_ levels, and blood cadmium concentrations with RCC. We categorized 5mdC (%), plasma folate and vitamin B_12_ levels, and blood cadmium concentrations into tertile based on the control distribution, and the lowest tertile was defined as the reference group. We also treated each tertile as an ordinal variable to test for a linear trend of OR in each stratum. To conduct interaction analysis, we categorized 5mdC (%), plasma folate and vitamin B_12_ levels, and blood cadmium concentrations at the median based on the control distribution and created a combined variable. This combination variable was examined for its association with RCC in the logistic regression models. We tested the multiplicative interaction between the two variables by using the product term in the logistic regression model. The synergy index was used to assess the additive interaction between the two variables [24]. All data were analyzed using the SAS software (version 9.4; Cary, NC, USA). Two-sided *p* < 0.05 was considered statistically significant.

## 3. Results

Table 1 shows the sociodemographic characteristics, lifestyle, and disease history of the RCC patients and non-RCC controls. There were no significant differences in the distribution of age, sex, and educational level between the 174 RCC patients and 673 controls in this study. A significantly higher percentage of RCC patients had body mass index (BMI) classified as overweight and obese than the control group. Additionally, 40.23% of RCC patients reported former and current cigarette smoking, which was relatively higher than the controls. The odds of having cumulative cigarette smoking ≥ 21 pack years were 1.64-fold greater among RCC patients compared to controls. In addition, the odds of frequent and occasional alcohol, tea, and coffee consumption were significantly decreased among RCC patients than controls. The proportion of RCC patients with a diagnosed history of diabetes, hypertension, and chronic kidney disease was significantly higher among patients with RCC than that among controls.

Associations of global DNA methylation marker-5mdC (%), plasma folate and vitamin B_12_ levels, and blood cadmium concentrations with RCC are shown in Table 2. The odds of higher 5mdC (%) (≥3.80) significantly decreased by 84% for the RCC patients than for the controls (multivariate adjusted OR (95% CI): 0.16 (0.09–0.30)). A significant decreasing trend was also observed between the elevated 5mdC (%) and RCC. Similarly, we observed that the odds of higher levels of plasma folate and vitamin B_12_ were significantly decreased among RCC patients compared to the controls, which were also in a dose–response manner. Conversely, we observed increased odds of higher concentrations of blood cadmium (≥1.67 μg/L) among RCC patients compared to the controls (multivariate adjusted OR (95% CI): 5.13 (2.92–9.02)).

**Figure 2:**
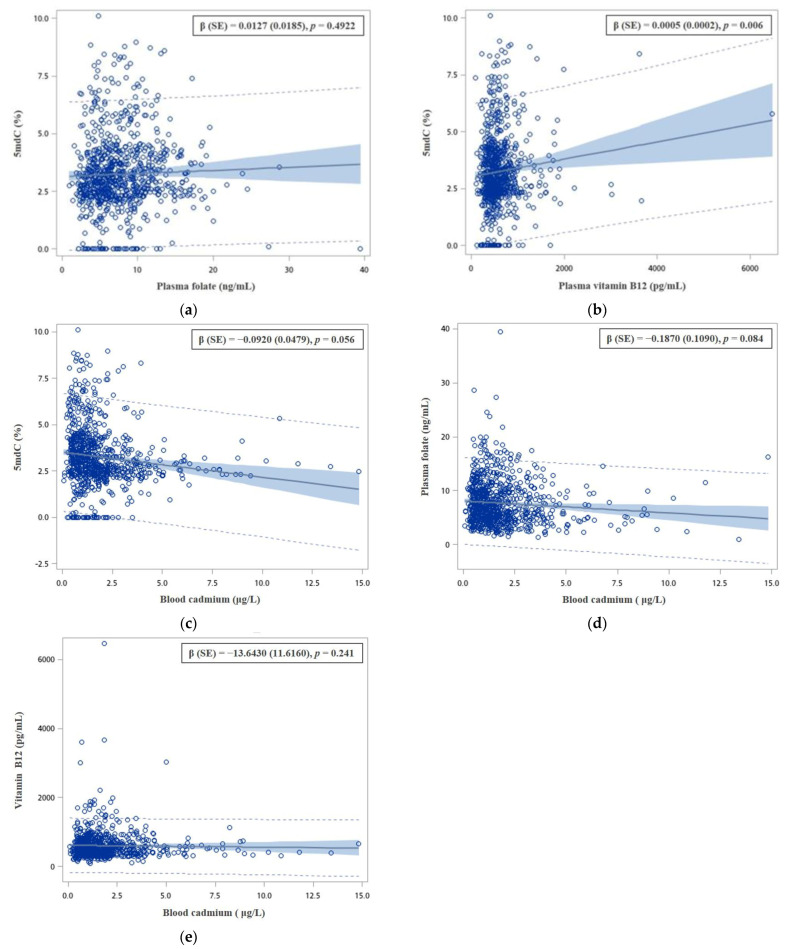
The association between (**a**) plasma folate level and 5mdC (%); (**b**) plasma vitamin B_12_ level and 5mdC (%); (**c**) blood cadmium concentration and 5mdC (%); (**d**) blood cadmium concentration and plasma folate level; (**e**) blood cadmium concentration and plasma vitamin B_12_ level. Coefficient (β) and standard error (SE) were estimated by multivariable linear regression models, adjusted for age, sex, educational levels, BMI, cumulative cigarette smoking, alcohol, tea, and coffee consumption, and diagnosed history of diabetes malleus, hypertension, and chronic kidney disease.

The associations of 5mdC (%), plasma folate and vitamin B_12_ levels, and blood cadmium concentrations are shown in Figure 2. We observed that plasma B_12_ levels were significantly positively associated with 5mdC (%), but plasma folate levels were not associated with 5mdC (%). In addition, we found that an increase in blood cadmium concentrations was marginally associated with decreasing levels of 5mdC (%) and plasma folate; however, an increase in blood cadmium concentrations was not associated with plasma B_12_ levels.

To assess the differences in 5mdC (%) and blood cadmium concentrations among participants with different levels of plasma folate and vitamin B_12_, a combination variable was created according to the levels of plasma folate (7.39 ng/mL) and vitamin B_12_ (532 pg/mL). We observed that 5mdC (%) significantly decreased sequentially in the high folate/high vitamin B_12_, high folate/low vitamin B_12_ or low folate/high vitamin B_12_, and low folate/low vitamin B_12_ groups. In addition, the 5mdC (%) of RCC patients in the three groups was significantly lower than that in the control group. However, these patterns were not observed for the comparisons of blood cadmium concentrations (Supplementary Table S2).

As blood cadmium concentrations and vitamin B_12_ levels have opposite effects on 5mdC (%), and blood cadmium concentrations were negatively associated with plasma folate levels, the interaction analysis between blood cadmium, plasma folate, and vitamin B12 on RCC was conducted. The combined effects of 5mdC (%), plasma folate, vitamin B_12_, and blood cadmium levels on RCC are presented in Table 3. For plasma folate levels > 7.39 ng/mL and 5mdC > 3.16 % as the reference group, the OR of RCC increased with exposure to an increasing number of risk factors (i.e., none, one, or both risk factors). The OR of RCC increased significantly with increasing risk factors for all combinations in a dose-dependent manner. The odds of plasma folate levels ≤ 7.39 ng/mL and 5mdC ≤ 3.16 % were 11.86-fold (95% CI: 5.27–26.65) greater among RCC patients compared to the controls after multivariable adjustment. Additionally, we observed that plasma folate was multiplicatively interacting significantly with 5mdC (%) (*p*_Interaction_ < 0.01) to increase the OR of RCC. Plasma folate levels also significantly additively interacted (S index = 3.15 (1.39–7.13)) with blood cadmium concentrations to elevate the OR for RCC. The odds of plasma folate levels ≤ 7.39 ng/mL and blood cadmium concentrations > 1.27 μg/L were 8.15-fold (95% CI: 1.39–7.13) greater among RCC patients compared to the controls after multivariable adjustment. No interactions were observed between vitamin B_12_ levels, 5mdC, and blood cadmium concentrations on RCC.

## 4. Discussion

In this study, we observed the inverse associations of the global DNA methylation marker-5mdC (%), plasma folate, and vitamin B_12_ levels with RCC. We also found that plasma vitamin B_12_ levels were positively associated with 5mdC (%), whereas blood cadmium concentrations were negatively associated with 5mdC (%). Additionally, low plasma folate levels tended to multiplicatively interact with low 5mdC (%) to increase the OR of RCC. Low plasma folate levels significantly additively interacted with high blood cadmium concentrations to elevate the OR of RCC.

Although a previous study showed that global DNA methylation varies with age, sex, BMI, and lifestyle factors, such as diet and cigarette smoking [25], our study observed an association between low 5mdC (%), representing global DNA hypomethylation, and an increased risk of RCC after controlling for age, sex, BMI, and lifestyle factors. This is consistent with previous studies that found that global DNA hypomethylation is related to RCC [20] and breast cancer [26]. The possible mechanism underlying our observed association is that genomic DNA hypomethylation is thought to have an important effect on tumors by inducing chromosomal instability, reactivation of transposable elements, and loss of imprinting [16]. Global DNA hypomethylation may also lead to activation of oncogenes that contribute to proliferation, differentiation, and cancer transformation [17]. However, another study did not support the finding that global DNA hypomethylation was associated with increased breast cancer risk [27]. Therefore, the association of 5mdC (%) as a marker of global DNA methylation with various cancers requires further research.

The present study observed that 5mdC (%) decreased as blood cadmium concentrations increased. A previous study also found a similar association that global DNA methylation in cord blood decreased with an increase in maternal cadmium intake assessed by the food frequency questionnaire [28]. In addition, chronic exposure to very low levels of cadmium in rats was found to cause persistent damage to the kidneys as well as increases in cell proliferation and global DNA hypomethylation [21]. This may be due to cadmium exposure increasing reactive oxygen species levels in the body and interrupting DNA methylation [29]. In addition, global DNA methylation levels significantly decreased with increasing cadmium concentration and exposure time, possibly due to increased oxidative DNA damage and downregulated expression of DNA methyltransferases 3a and 3b [30].

One-carbon metabolism requires nutrients, including folate and B vitamins, and involves the coordination of the folate and methionine cycles to produce S-adenosylmethionine, a universal methyl donor that significantly increases global DNA methylation [10]. In the present study, we found that low plasma folate and vitamin B_12_ levels increased the risk of RCC. This is consistent with a previous study that found serum folate was inversely associated with the risk of RCC [31]. Additionally, evidence has suggested that plasma homocysteine concentrations were significantly negatively correlated with the concentrations of serum folate and vitamin B_12_ [31,32,33]. However, our study was not able to confirm this association, as we did not measure plasma homocysteine levels. Additionally, we found that an increased level of plasma vitamin B_12_ was associated with increasing the global DNA methylation markers-5mdC (%). An increased level of plasma folate level was also positively associated with 5mdC (%), though this association was not significant. Furthermore, we observed that low plasma folate significantly multiplicatively interacted with low 5mdC (%) to increase the OR of RCC. A mechanism proposed for this combined effect may be because folate is involved in one-carbon metabolism, which is a prerequisite for processes related to carcinogenesis including DNA hypomethylation [34]. Furthermore, these bioactive food components play a role in increasing DNA methylation patterns to prevent cancer [35].

In the present study, the OR of RCC increased due to a significant additive interaction between low plasma folate concentrations and high blood cadmium levels. This is consistent with previous research showing that cadmium exposure-induced inhibition of DNA methyltransferase was greater in rats fed a methyl-deficient diet (deficient in choline and folate) compared to that of in controls [36]. These results provide evidence that the carcinogenic effects of cadmium may be mediated in part through aberrant DNA methylation in folate-deficient conditions.

This study has some limitations. As this study was a case–control study, it cannot be ruled out that the association of environmental factors with RCC may be a consequence rather than a cause of RCC. This study used only one sample to assess plasma folate, vitamin B_12_, and blood cadmium concentrations. Thus, these measurements would be reliable only if all patients included had a stable lifestyle and constant metabolism. Future research should include multiple urine samples to further improve exposure assessment. Additionally, detailed information on folate and supplement intake were not obtained in the present study. However, a previous study has indicated that measuring folate concentration in plasma was highly related to folate intake [37]. Because the sample size was relatively small, the significance of the findings should be interpreted with caution. Further studies with larger sample sizes are necessary to verify these results. Nevertheless, these findings are crucial for understanding the potential factors associated with cadmium-related RCC.

## 5. Conclusions

To the best of our knowledge, this study is the first to identify significant additive interactions between low plasma folate levels and high blood cadmium levels that increase the OR of RCC. In addition, low plasma folate levels significantly multiplicatively interacted with low 5mdC (%) to increase the OR of RCC. This study provides suggestive evidence that plasma folate may modify the associations between global DNA methylation or environmental factors, such as blood cadmium, and RCC.

## Figures and Tables

**Table 1:** Sociodemographic characteristics, lifestyle, and disease histories of RCC cases and non-RCC controls.

Characteristic	RCC Cases *n* (%)	Controls *n* (%)	*p*-Value ^a^	Age-Sex Adjusted OR (95% CI)
Age (years)	58 (49, 69) ^b^	59 (51, 71) ^b^	0.22	0.99 (0.98–1.01) ^c^
Sex			0.15	
Male	121 (69.54)	429 (63.74)		1.00 ^d^
Female	53 (30.46)	244 (36.26)		0.77 (0.54–1.11)
Education			0.10	
Elementary school or below	37 (21.26)	129 (19.23)		1.00
Junior/Senior high school	71 (40.80)	228 (33.98)		0.92 (0.57–1.47)
College or above	66 (37.94)	314 (46.80)		0.56 (0.34–0.93) **
BMI (kg/m^2^)			<0.01	
Normal (≤23.99)	70 (40.23)	456 (67.76)		1.00
Overweight (24.0–26.99)	53 (30.46)	125 (18.57)		2.71 (1.79–4.10) **
Obese (≥27.0)	51 (26.31)	92 (13.67)		3.55 (2.32–5.45) **
Cigarette smoking			0.03	
Non-smoker	104 (59.77)	462 (68.65)		1.00
Former or current smoker	70 (40.23)	211 (31.35)		1.42 (0.96–2.10) ^+^
Cumulative cigarette smoking (pack years)			0.02	
0	104 (61.18)	456 (67.76)		1.00
0 < pack year ≤ 21	30 (17.65)	125 (18.57)		1.31 (0.80–2.14)
Pack year > 21	36 (21.18)	92 (13.67)		1.64 (1.01–2.66) *
Alcohol consumption			<0.01	
Never	132 (75.86)	400 (59.44)		1.00
Occasional or frequent	42 (24.14)	273 (40.56)		0.39 (0.26–0.58) **
Tea consumption			<0.01	
Never	84 (48.28)	222 (32.99)		1.00
Occasional or frequent	90 (51.72)	451 (67.01)		0.50 (0.35–0.70) **
Coffee consumption			0.01	
Never	107 (61.49)	316 (46.95)		1.00
Occasional or frequent	67 (38.51)	357 (53.05)		0.54 (0.39–0.77) **
Diabetes mellitus			<0.01	
No	139 (79.89)	617 (92.09)		1.00
Yes	35 (20.11)	53 (7.91)		3.16 (1.96–5.09) **
Hypertension			<0.01	
No	94 (54.02)	502 (74.93)		1.00
Yes	80 (45.98)	168 (25.07)		2.84 (1.97–4.09) **
Chronic kidney disease			<0.01	
No	135 (77.59)	591 (87.82)		1.00
Yes	39 (22.41)	82 (12.18)		2.32 (1.49–3.63) **

Abbreviation: BMI, body mass index; OR, odds ratio; CI, confidence interval. Data of two controls for educational level, that of four RCC patients for cumulative cigarette smoking, that of three controls for diabetes mellitus, and that of three controls for hypertension status were unavailable. ^a^ *p*-values were tested using the Wilcoxon rank-sum test for age and χ^2^ test for the rest of categorical variables. ^b^ Data summarized as median (first and third quartile). ^c^ Sex-adjusted OR (95%CI). ^d^ Age-adjusted OR (95% CI). ^+^ 0.05 ≤ *p* < 0.1, * *p* < 0.05, ** *p* < 0.01.

**Table 2:** Associations of 5mdC (%), plasma folate and vitamin B_12_ levels, and blood cadmium concentrations with RCC.

Variables	RCC Cases *n* (%)	Controls *n* (%)	Age-Sex Adjusted OR (95% CI)	Multivariate Adjusted OR (95% CI) ^b^
5mdC (%)	2.47 (2.07, 3.17) ^a^	3.83 (4.90, 7.26) ^a,^**		
≤2.70	109 (62.64)	225 (33.43)	1.00 ^#^	1.00 ^#^
2.71–3.79	47 (27.01)	244 (33.28)	0.43 (0.29–0.64) **	0.53 (0.33–0.84) **
≥3.80	18 (10.74)	224 (33.28)	0.17 (0.10–0.28) **	0.16 (0.09–0.30) **
Plasma folate levels (ng/mL)	4.95 (3.29, 7.34) ^a^	7.39 (5.18, 10.20) ^a,^**		
≤5.85	113 (64.94)	226 (33.58)	1.00 ^#^	1.00 ^#^
5.86–9.20	33 (18.97)	223 (33.14)	0.30 (0.19–0.46) **	0.27 (0.16–0.45) **
≥9.21	28 (16.09)	224 (33.28)	0.25 (0.16–0.40) **	0.26 (0.15–0.44) **
Plasma vitamin B_12_ levels (pg/mL)	468 (360, 655) ^a^	532 (410, 715) ^a,^**		
≤445	79 (45.40)	225 (33.43)	1.00 ^#^	1.00 ^#^
446–621	48 (27.58)	224 (33.28)	0.61 (0.41–0.92) *	0.59 (0.37–0.95) *
≥622	47 (27.01)	224 (33.28)	0.63 (0.42–0.95) *	0.57 (0.35–0.94) *
Blood cadmium concentrations (μg/L)	1.86 (1.16, 2.86) ^a^	1.27 (0.78, 2.08) ^a,^**		
≤0.92	30 (17.24)	236 (35.07)	1.00 ^#^	1.00 ^#^
0.93–1.66	44 (25.29)	218 (32.39)	1.74 (1.05–2.88) *	2.17 (1.21–3.88) **
≥1.67	100 (57.49)	219 (32.54)	3.97 (2.52–6.26) **	5.13 (2.92–9.02) **

Abbreviation: OR, odds ratio; CI, confidence interval. ^a^ Data summarized as median (first and third quartile); and Wilcoxon rank-sum test was conducted for comparison. ^b^ Multivariate ORs were adjusted for age, sex, educational levels, BMI, cumulative cigarette smoking, alcohol, tea, and coffee consumption, and diagnosed history of diabetes malleus, hypertension, and chronic kidney disease. * *p* < 0.05, ** *p* < 0.01. ^#^ *p* < 0.05 for trend test.

**Table 3:** Combined effects of 5mdc (%), plasma folate and vitamin B_12_, and blood cadmium levels on RCC.

Variables 1	Variables 2	Cases/Controls	Age-sex Adjusted OR (95% CI)	Multivariate Adjusted OR (95% CI) ^a^
Plasma folate levels (ng/mL)	5mdC (%)			
>7.39	>3.16	9/176	1.00 ^#^	1.00 ^#^
>7.39	≤3.16	32/159	4.01 (1.85–8.68) **	3.79 (1.60–8.97) **
≤7.39	>3.16	36/159	4.49 (2.09–9.63) **	4.61 (1.99–10.70) **
≤7.39	≤3.16	97/179	10.78 (5.24–22.19) **	11.86 (5.27–26.65) **
			S = 1.50 (0.95–2.39)	S = 1.70 (0.97–2.96)
			*p*_Interaction_ < 0.01	*p*_Interaction_ < 0.01
Plasma vitamin B_12_ levels (pg/mL)	5mdC (%)			
>532	>3.16	20/173	1.00 ^#^	1.00 ^#^
>532	≤3.16	45/161	2.45 (1.38–4.34) **	2.32 (1.09–4.52) *
≤532	>3.16	25/162	1.32 (0.71–2.47)	1.50 (0.74–3.05)
≤532	≤3.16	84/177	4.01 (2.35–6.86) **	4.62 (2.46–8.66) **
			S = 1.70 (0.80–3.62)	S = 1.99 (0.83–4.76)
			*p*_Interaction_ = 0.44	*p*_Interaction_ = 0.25
5mdC (%)	Blood cadmium concentrations (μg/L)			
>3.16	≤1.27	14/200	1.00 ^#^	1.00 ^#^
>3.16	>1.27	31/135	3.46 (1.77–6.76) **	4.54 (2.10–9.78) **
≤3.16	≤1.27	39/144	3.79 (1.98–7.26) **	3.89 (1.88–8.03) **
≤3.16	>1.27	90/194	6.84 (3.76–12.45) **	8.16 (4.10–16.24) **
			S = 1.11 (0.69–1.80)	S = 1.11 (0.64–1.93)
			*p*_Interaction_ = 0.31	*p*_Interaction_ = 0.84
Plasma folate levels (ng/mL)	Blood cadmium concentrations (μg/L)			
>7.39	≤1.27	15/174	1.00 ^#^	1.00 ^#^
>7.39	>1.27	26/161	1.93 (0.98–3.78) ^+^	1.98 (0.91–4.31) ^+^
≤7.39	≤1.27	38/170	2.49 (1.31–4.72) **	2.29 (1.10–4.75) *
≤7.39	>1.27	95/168	6.47 (3.60–11.61) **	8.15 (4.07–16.29) **
			S = 2.26 (1.16–4.42)	S = 3.15 (1.39–7.13)
			*p*_Interaction_ = 0.34	*p*_Interaction_ = 0.05
Plasma vitamin B_12_ levels (pg/mL)	Blood cadmium concentrations (μg/L)			
>532	≤1.27	15/174	1.00 ^#^	1.00 ^#^
>532	>1.27	50/160	3.71 (2.00–6.89) **	4.89 (2.31–10.32) **
≤532	≤1.27	38/170	2.40 (1.27–4.56) **	3.07 (1.44–6.54) **
≤532	>1.27	71/163	4.83 (2.66–8.77) **	7.86 (3.76–16.42) **
			S = 0.93 (0.56–1.56)	S = 1.15 (0.66–2.01)
			*p*_Interaction_ = 0.63	*p*_Interaction_ = 0.43

Abbreviation: OR, odds ratio; CI, confidence interval; S, synergy index; *p*_Interaction_, *p* value tested for the product term. ^a^ Multivariate ORs were adjusted for age, sex, educational levels, BMI, cumulative cigarette smoking, alcohol, tea, and coffee consumption, and diagnosed history of diabetes malleus, hypertension, and chronic kidney disease. ^+^ 0.05 ≤ *p* < 0.1, * *p* < 0.05, ** *p* < 0.01. ^#^ *p* < 0.05 for trend test.

## Data Availability

The data presented in this study are available on request from the corresponding author. The data are not publicly available due to privacy or ethical restrictions.

## Supplementary Materials

The following supporting information can be downloaded at: https://www.mdpi.com/article/10.3390/nu15040937/s1, Table S1: Validity and reliability of the methods for determining plasma folate and vitamin B_12_ levels as well as blood cadmium concentrations; Table S2: Comparison of 5mdc (%) and blood cadmium concentrations between RCC cases and controls stratified by a combination of plasma folate and vitamin B_12_ levels.
