# Supplementary material for: Possible Combined Effects of Plasma Folate Levels, Global DNA Methylation, and Blood Cadmium Concentrations on Renal Cell Carcinoma
Source: Nutrients. 2023 Feb 13;15(4):937. doi: 10.3390/nu15040937 (PMC9959822; doi:10.3390/nu15040937)
Supplement: Supplementary file 1 [file nutrients-15-00937-s001.zip › nutrients-2195350-supplementary.pdf]

nutrients

MDPI

Supplementary Materials

Possible Combined Effects of Plasma Folate Levels, Global DNA Methylation, and Blood Cadmium Concentrations on Renal Cell Carcinoma

Chao-Yuan Huang [1], Wei-Jen Chen [2], Hui-Ling Lee [3], Ying-Chin Lin [4,5,6], Ya-Li Huang [7], Horng-Sheng Shiue [8], Yeong-Shiau Pu [1] and Yu-Mei Hsueh [4,7,*]

[1] Department of Urology, National Taiwan University Hospital, College of Medicine National Taiwan University, Taipei City 110, Taiwan; cyhuang0909@ntu.edu.tw (C.-Y.H.); yspu@ntu.edu.tw (Y.-S.P.)
[2] Department of Medicine, Section of Epidemiology and Population Sciences, Baylor College of Medicine, Houston, TX 77030, USA; wei-jen.chen@bcm.edu
[3] Department of Chemistry, Fu Jen Catholic University, New Taipei City 242, Taiwan; 076308@mail.fju.edu.tw
[4] Department of Family Medicine, Wan Fang Hospital, Taipei Medical University, Taipei City 110, Taiwan; green1990@tmu.edu.tw
[5] Department of Family Medicine, School of Medicine, College of Medicine, Taipei Medical University, Taipei City 110, Taiwan
[6] Department of Occupational Medicine, Wan Fang Hospital, Taipei Medical University, Taipei City 110, Taiwan
[7] Department of Public Health, School of Medicine, College of Medicine, Taipei Medical University, Taipei City 110, Taiwan; ylhuang@tmu.edu.tw
[8] Department of Chinese Medicine, College of Medicine, Chang Gung University, Taoyuan City 333, Taiwan; hongseng@ms1.hinet.net
* Correspondence: ymhsueh@tmu.edu.tw; Tel.: +886-2736-1661 (ext. 6513)

Supplementary Table S1. Validity and reliability of the methods for determining plasma folate and vitamin B_{12} levels as well as blood cadmium concentrations.

	Instrument	Detection Limit	Recovery Rate	Standard Reference Materials (SRM)	Coefficient of Variance (CV)
**Plasma folate**	Radioassay kit (Bio-Rad, Richmond, CA) and the 1470 Wizard series gamma counter	0.6 ng/mL	-	-	9.3%
**Plasma vitamin B_{12}**		75 pg/mL	-	-	6.8%
**Blood cadmium**	Inductively coupled plasma mass spectrometry	0.07 μg/L	100 ± 20%	SRM (Seronorm Trace Elements Whole Blood L-2 (Lot 1103129)) certificate value 5.8 μg/L (range 5.4–6.2 μg/L); 6.1 ± 0.5 μg/L in our system	< 5%

Supplementary Table S2. Comparison of 5mdC (%) and blood cadmium concentrations between RCC cases and controls stratified by a combination of plasma folate and vitamin B_{12} levels.

	Overall	RCC Cases	Controls
	High/high group for plasma folate and vitamin B_{12} (n = 237)		
**5mdC (%)**	3.16 (2.46, 4.24) [a]	2.27 (1.81, 2.62) [c,e]	3.28 (2.54, 4.34) [d,e]
**Blood cadmium concentrations (µg/L)**	1.30 (0.84, 2.20)	1.80 (1.12, 2.20) [f]	1.23 (0.78, 2.22) [f]
	Low/high or high/low groups for plasma folate and vitamin B_{12} (n = 301)		
**5mdC (%)**	3.05 (2.33, 4.05) [b]	2.54 (2.10, 3.47) [c,g]	3.20 (2.50, 4.16) [g]
**Blood cadmium concentrations (µg/L)**	1.32 (0.80, 2.18)	2.00 (1.32, 2.98) [h]	1.24 (0.74, 1.80) [h]
	Low/low group for plasma folate and vitamin B_{12} (n = 309)		
**5mdC (%)**	2.83 (2.28, 3.75) [a,b]	2.45 (2.10, 3.06) [i]	3.04 (2.40, 3.97) [d,i]
**Blood cadmium concentrations (µg/L)**	1.50 (0.92, 2.30)	1.86 (1.16, 3.28)	1.32 (0.82, 2.14)

A combination of high plasma folate (> 7.39 ng/mL) and high vitamin B_{12} (> 532 pg/mL) was defined as the high/high group; a combination of low plasma folate (≤ 7.39 ng/mL) and high vitamin B_{12} (> 532 pg/mL) was defined as the low/high group; a combination of high plasma folate (> 7.39 ng/mL) and low vitamin B_{12} (≤ 532 pg/mL) was defined as the high/low groups; and a combination of low plasma folate (≤ 7.39 ng/mL) and low vitamin B_{12} (≤ 532 pg/mL) was defined as the low/low group. Values are expressed as median (first quartile, third quartile). Kruskal-Wallis and Wilcoxon tests were conducted to compare the 5mdC (%) and blood cadmium concentrations between groups. The same letters indicate a significant difference (p-value < 0.05) between the two groups.
